# Multidisciplinary lifestyle intervention to manage pancreatic cancer-related cachexia: a case report

**DOI:** 10.2144/fsoa-2020-0165

**Published:** 2020-11-12

**Authors:** Alice Avancini, Ilaria Trestini, Daniela Tregnago, Alessandro Cavallo, Marco Bragato, Clelia Bonaiuto, Massimo Lanza, Michele Milella, Sara Pilotto

**Affiliations:** 1 Department of Medicine, Biomedical, Clinical & Experimental Sciences, University of Verona Hospital Trust, Verona 37134, Italy; 2 Department of Medicine, Medical Oncology, University of Verona Hospital Trust, Verona 37134, Italy; 3 Department of Neurosciences, Biomedicine & Movement Sciences, University of Verona, Verona 37134, Italy

**Keywords:** cachexia, exercise, lifestyle intervention, metastatic disease, nutrition, pancreatic cancer, psychological support

## Abstract

Pancreatic cancer remains an aggressive disease, with a poor prognosis and a high risk of incurring into cachexia. Supportive care, such as exercise, nutritional and psychological support, may be effective in reducing functional loss, psychological distress and improving nutritional status. We report the effect of 12 weeks of multimodal lifestyle intervention in a 55-year-old female, diagnosed with unresectable body/tail pancreatic cancer and metastasis in the liver, bone, lymph node and lung, to counteract cachexia. The multimodal program resulted safe and feasible. Over 12 weeks, considerable improvements were found in body weight, health-related physical fitness, nutritional status, distress scores, anxiety and depression levels. These findings highlight the potential role of integrated supportive interventions to manage metastatic cancer and cancer-induced cachexia.

## Lay abstract

Pancreatic cancer has an unfavorable prognosis and a high risk of developing cachexia. Cachexia is a complex syndrome, manifesting with weight and muscle mass loss, usually leading to important metabolic disorders. To date, no effective treatments are available to reverse cancer-related cachexia, that it is also associated with a reduced probability of survival. In this report, we describe the benefits of an integrated intervention, including exercise, nutritional and psychological counseling, as a strategy to support a cachectic patient affected by metastatic pancreatic cancer.

Although relatively uncommon (2.5% of all cancers), pancreatic cancer (PC) remains a lethal malignancy, with a ratio mortality/incidence of 94% and a 5-year survival rate of only 9% [1]. To date, effective therapies are available to improve prognosis and relieve patient’s symptoms. Surgery with curative intent represents the main opportunity for ‘cure’, even though the vast majority (∼85%) of patients presents with unresectable disease [2]. Chemotherapy (mostly gemcitabine- and fluorouracil-based polychemotherapy combinations) has a definite impact on survival in both resectable and advanced disease, and radiation therapy is used mainly to treat locally advanced, inoperable disease [2].

PC patients are at high-risk of cachexia, a multifactorial syndrome characterized by an ongoing loss of skeletal muscle mass that cannot be fully reversed by conventional nutritional support and leads to progressive functional impairment [3]. Currently, no standard treatments are available to contrast the progression of cancer cachexia [4]. In light of its dismal prognosis, advanced PC treatment remains palliative in nature, and managing patients’ physical function and preserving their quality of life (QoL) is at least as important as extending survival [5]. Supportive multimodal care, physical exercise, dietary guidance and psychological support, have established efficacy to counteract many cancer- and treatment-related side effects [6,7] and might represent a useful approach to treat or prevent cancer-induced cachexia. Exercise is a potent body modulator, able to increase cardiorespiratory fitness, strength and muscle mass, which, in turn, represent independent predictors of survival in cancer; moreover, increased physical fitness may counteract some disabling cancer symptoms, such as fatigue, nausea, pain, anxiety and depression [6]. Food intake optimization has been recognized as a crucial approach in the treatment of PC patients, considering that they frequently suffer from malnutrition and experience a reduced food intake due to several reasons (e.g., loss of appetite, anorexia, maldigestion and malabsorption, vomiting, nausea) [8]. Therefore, increasing energy intake and protein balance with a personalized nutritional counseling can improve body composition, prevent weight loss and manage some cancer- and treatment-related side effects [3]. Finally, PC patients usually report a high level of distress, which can exacerbate symptoms burden, impair QoL and interfere with medical treatments [9]. Psychotherapy, including cognitive–behavioral therapy, problem-solving therapy or mindfulness-based approaches, for example, has demonstrated to reduce anxiety and depression levels in advanced cancer patients [10]. Moreover, psychological support can be effective to manage cancer-related fatigue, reduce fear and improve global well-being [10].

Exercise, nutritional and psychological support complement each other, possibly resulting in synergistic potentiation of the expected clinical benefit by the appropriate combination of these interventions, particularly in a complex and aggressive disease such as PC. Nevertheless, safety and feasibility of an integrated, multimodal approach in advanced cancer with cachexia is still a relatively unexplored area. Here, we report the results of a three-month supervised, integrated supportive intervention, carried out by a dedicated multidisciplinary team (Focus On Research and CarE - FORCE - team), including exercise, nutritional counseling and psychological support in an advanced, metastatic PC patient with cachexia undergoing second- and third-line chemotherapy.

## Case presentation

**Figure 1.**
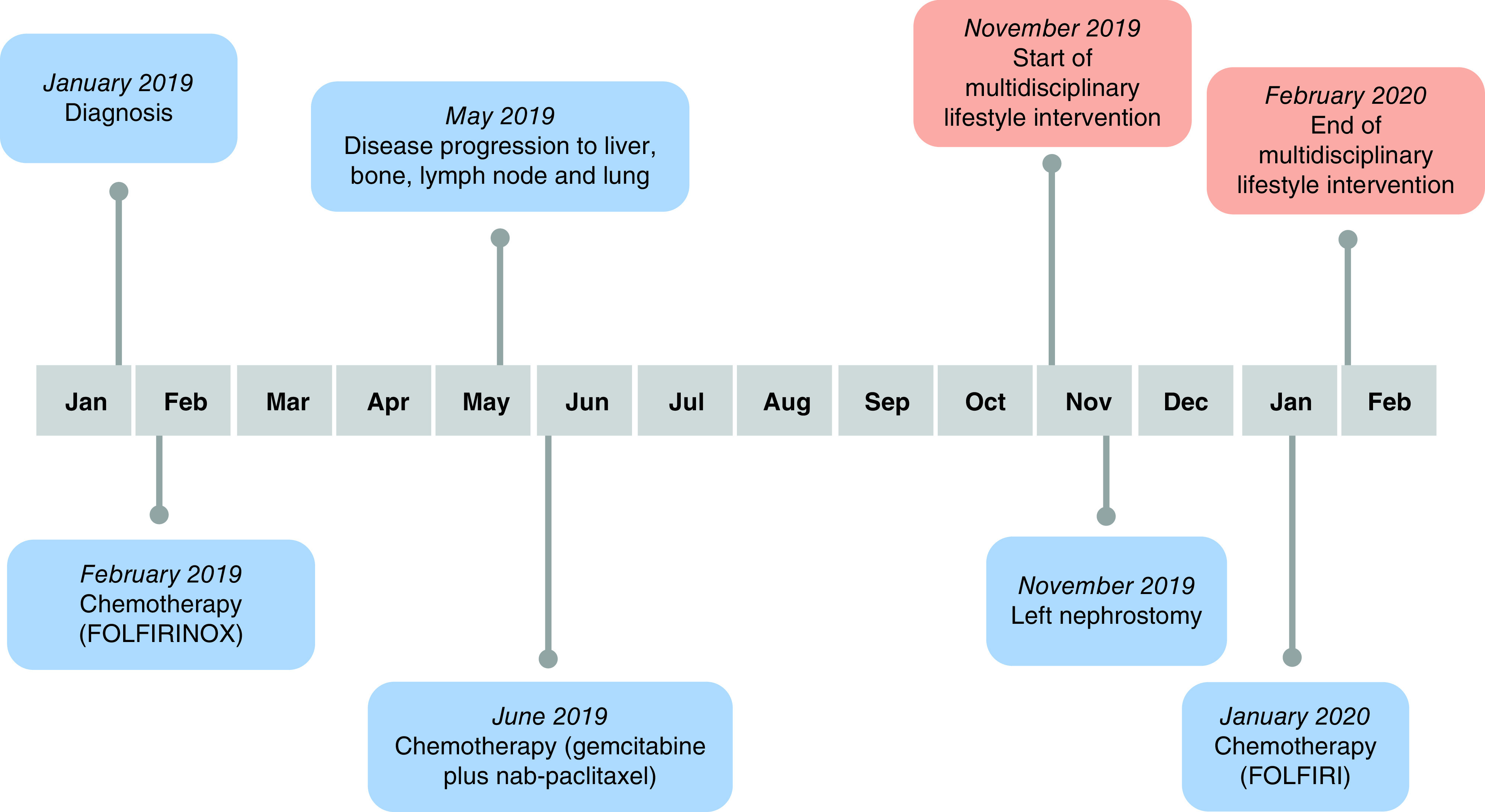
Timeline of disease status and multidisciplinary lifestyle intervention.

A 55-year-old woman presented in January 2019 with unintentional weight loss and abdominal pain. Computer tomography of the abdomen and subsequent ultrasound-guided fine-needle aspiration biopsy led to the diagnosis of an unresectable body/tail PC (stage 3 according to Tumor, Node, Metastasis [TNM] classification). She underwent chemotherapy with a combination of fluorouracil, leucovorin, irinotecan and oxaliplatin (FOLFIRINOX), but after three months, disease progressed with appearance of liver and bone metastases (Figure 1). Second-line chemotherapy was then started with gemcitabine/nab-paclitaxel in June 2019, resulting in disease stabilization and temporary clinical benefit. In November 2019, disease progressed further, involving abdominal lymph nodes, liver, lung and bones, and resulting in a malignant upper left urinary tract obstruction, which required invasive palliation by nephrostomy.

At the time of observation by the FORCE team (November 2019), with a weight loss of 21.4% over the past 6 months without starvation the patient was considered cachectic (according to EPCRC criteria) [11] and the Eastern Cooperative Oncology Group (ECOG) performance status was 1. Despite the current lack of survival benefit in this setting, therapeutic options, including best supportive care, were discussed with the patient and she was candidate to third-line palliative chemotherapy (FOLFIRI) (Figure 1). At the same time, she was offered to participate into an integrated three-month multimodal program, including exercise, nutrition and psychological intervention. The three-month period was considered an adequate time frame to achieve a meaningful change in weight, also considering the prognostic expectation at this disease stage [12].

The study was conducted according to the Declaration of Helsinki, the Good Clinical Practice and was reported following case report (CARE) guidelines [13]. The authors obtained patient’s consent for publication of clinical data. The patient’s personal details were anonymized.

### Multimodal intervention

#### Exercise

An individual exercise program based on the American College of Sports Medicine guidelines [6] and supervised by an experienced kinesiologist, was conducted with the aim to improve cardiorespiratory fitness and increase muscle mass and strength. Baseline evaluation included cardiorespiratory fitness, strength, flexibility (Table 1) and complete medical history. A bi-weekly program was implemented, with each session lasting 60 min and including in order: warm-up, aerobic exercises, strength activities and cool-down. Fifteen-min warm-up and cool-down phases comprised dynamic and static flexibility exercises, respectively. The load of aerobic activity, consisting in cycling and walking ergometer, was slowly increased from 10 min at the beginning to 25 min at the end of the program, with an intensity level of 3–5 on the CR10 Borg Scale of perceived exertion. Resistance training included six exercises with bodyweight and resistance bands (Thera-Band, Hygenic Corp., OH, USA), covering major functional lower- and upper-body muscle groups. Each strength exercise was performed at 3–5 on the CR10 Borg Scale of perceived exertion, in two-three sets of 8–12 repetitions, which were progressively increased during the training program.

**Table 1.** Absolute scores of health-related physical fitness and nutritional parameters.

Measure	At baseline	Postintervention
**Resting blood pressure and heart rate**		
– Resting systolic blood pressure (mmHg)	103	109
– Resting diastolic blood pressure (mmHg)	70	61
– Resting heart rate (bpm)	68	61
**6-min walking test (m)**	416.0	525.6
– Final heart rate	87	86
– RPE	3.0	3.0
**Handgrip strength (kg)**		
– Right arm	22	24
– Left arm	22	23
– RPE	4.0	5.5
**Chair sit and reach (cm)**		
– Right leg	0.0	0.0
– Left leg	−2.0	+2.0
**Back scratch (cm)**		
– Right arm (upper)	+3.5	+5.5
– Left arm (upper)	+4.0	+3.0
**Anthropometric parameters**		
– Usual weight (kg)	63.0	
– Usual BMI (kg/m^2^)	23.1	
– 6-month weight loss (%)	21.4	
– Body weight (kg)	49.0	53.2
– BMI (kg/m^2^)	18.0	19.5
– Waist (cm)	67.1	70.5
– Hip (cm)	89.3	92.0
– Waist–hip ratio	0.8	0.8
**Body composition**		
– Phase angle (degrees)	2.9	3.8
– Body cell mass (kg)	7.5	10.4
– Total body water (l)	20.8	21.0
– Fat mass (kg)	6.1	5.2
– Fat free mass (kg)	23.9	26.1
**NRS-2002 score**	3.0	2.0
**Dietary assessments**		
– Estimated energy requirements (kcal/day)	1836	
– Estimated protein requirements (g/kg/day)	1.5	
– Baseline energy intake (kcal/day)	1271	1874
– Baseline protein intake (g/kg/day)	0.8	1.4
**Nutritional impact symptoms**		
– Early satiety	Yes	No
– Dysphagia	Yes	No
– Loss of appetite	Yes	No
– Dysgeusia	No	No
– Oral mucositis	Yes	No
– Dyspepsia	Yes	No
– Xerostomia	Yes	No
– Nausea/vomiting	Yes	Yes
– Diarrhea	Yes	No
– Steatorrhea	Yes	No
– Abdominal bloating	Yes	No

NRS: Nutritional risk screening; RPE: Rate of perceived exertion.

#### Nutritional intervention

Nutritional intervention had the main objective to meet patient’s energy and protein requirements and to effectively manage disease- and treatment-related symptoms with a nutritional impact. Nutritional counseling was carried out bi-weekly, in presence, by a registered dietitian with experience in cancer care: intervention consisted in a personalized dietary prescription, including sample meal plans and suggested recipes, tailored to patient’s own eating patterns and preferences. Patient was invited to take more time to eat, increase the daily number of meals and snacks, and favor high-protein and -energy food. Total daily energy requirements were calculated at baseline by the Harris–Benedict equation, corrected by a factor of 1.5 [7], whereas daily protein requirement was set at 1.5 g/kg of actual body weight [7]. Since spontaneous oral intake was insufficient to cover needs, oral nutritional supplements, with high protein and calories content, were proposed [7]. Finally, pancreatic enzyme replacement therapy (PERT) was prescribed for the management of malabsorption symptoms: The initial PERT dose was 40,000 U Ph Eur and 25,000 U Ph Eur of lipase per meal and per snack, respectively. The patient was trained to take PERT during the meal and to adapt the dose based on meal size and fat content; clinical symptoms and the presence of steatorrhea were evaluated bi-weekly.

#### Psychological support

Weekly psychological support sessions were carried out, with each meeting lasting about 60 min. Using cognitive–behavioral therapy, the primary focus of psychological treatment was helping the patient to reduce anxiety, depression and distress levels. After baseline assessment, based on current guidelines [14], intervention started from behavioral reactivation, with the aim to implement patient’s daily living activities. Through cognitive restructuring, the existing dysfunctional and irrational thoughts were modified, promoting useful and functional ones. Moreover, a mindfulness-based approach was proposed to implement patient’s quality of sleep, reducing the nightly awakening and the ruminations. The sessions also incorporated progressive relaxation techniques and controlled breathing, to decrease muscle tension.

### Assessments

Outcome measures were assessed at baseline and at 12 weeks. At baseline, demographic information was self-reported, whereas clinical data were derived from the electronic patient data management system. Resting heart rate and blood pressure were evaluated before health-related fitness assessment, after 10 min of rest in the supine position.

#### Safety & feasibility

Safety was classified as intervention-related adverse events, occurring as a direct result of exercise or nutritional or psychological support and categorized according to the Common Terminology Criteria for Adverse Events (version 5.0). Feasibility was re-evaluated continuously during intervention, recording the adherence to each intervention, in other words, the number of sessions attended by the subject, compared with the total planned.

#### Health-related physical fitness

A series of tests were performed to evaluate the physical and functional capacity of the patient. A 6-min walking test was used to assess cardiorespiratory fitness, according to the American Thoracic Society guidelines [15]. The test consisted in walking in a 20-m hallway, with the goal to cover as much distance as possible in six minutes. Standardized encouragements were given and the time remaining was called out every minute. Muscular strength was evaluated with the handgrip strength test, using a hydraulic hand dynamometer (Model SH5001, Saehan Corporation, South Korea). The subject was sitting in a straight-backed chair with the feet flat on the floor, the shoulders in adducted and neutral position, and the wrist between 0–30 degrees extension and between 0–15 degrees ulnar deviation. For both arms, five tests were performed, and each voluntary contraction was kept for 2–4 s, with one-minute rest between the tests. The highest achieved value in each hand was reported [16]. Flexibility was evaluated for upper and lower limbs, using back scratch and chair sit and reach test, following the protocol proposed by Rickli and Jones [17]. Anthropometric parameters included BMI, obtained by the weight of the subjects divided by the square of her height and waist–hip ratio, derived by the ratio of waist and hip circumferences, according to standard procedures [18].

#### Nutritional assessments

Nutritional risk screening (NRS-2002) was adopted to evaluate nutritional risk through the following variables, referred to the previous week: weight loss, BMI, general conditions, amount of food intake, age and severity of the disease. According to the scoring protocol the patient is classified at nutritional risk (score equal or more than three) or not (score less than three) [19]. The dietitian collected the presence of symptoms potentially affecting patient’s feeding, such as early satiety, loss of appetite, dysgeusia, dyspepsia, chemotherapy-induced nausea and vomiting, xerostomia, and symptoms of malabsorption, including increased abdominal bloating or discomfort, excessive gas causing burping or flatulence, increased frequency, light color, floating, frothy, oily and/or foul-smelling feces. Energy intake was assessed by a 3-day 24-h dietary recall method (2 weekdays and one weekend day). The nutrient contents of foodstuffs and meals were analyzed by the Food Composition Table of National Institute for Research on Food and Nutrition. This energy intake was comparable with the patient’s optimal nutritional requirements. Inadequacy of energy intake was considered in the event of a current energy intake <60% of estimated requirements for more than 1–2 weeks, according to the most recent guidelines for nutrition in cancer patients of the European Society for Clinical Nutrition and Metabolism (ESPEN) [7]. Body composition was assessed using the NUTRILAB BIVA (Akern s.r.l., Florence, Italy), according to previously described procedures [20].

#### Psychological & patients reported outcomes

A series of questionnaires were proposed to evaluate psychological status, QoL and physical activity level. Psychological status was assessed using validated tools: Hospital anxiety and depression scale (HADS) and the Distress Thermometer (DT) [21]. HADS, is a self-reported questionnaire composed by 7-item regarding anxiety (HADS-A) and 7-item concerning depression (HADS-D) and reflects how the patient felt in the previous week. Both scales ranging from 0 to 21, and a score of 8–10 reflect borderline symptoms while scoring ≥10 indicates the presence of clinically relevant of anxiety and depression [21]. DT is a single-item question, in which on an 11-point numerical analogue scale the subject quantified her distress from 0 (no distress) to 10 (extreme distress). A score equivalent to or greater than 4 suggests a clinically significant level of distress [22]. The European Organization for Research and Treatment of Cancer Quality of Life Questionnaire (EORTC QLQ C-30) was used to assess the QoL. The EORTC QLQ C-30 is a 30 items scale that measures global health status (2 items), symptoms subscale (13 items: fatigue, pain, nausea, vomiting, dyspnea, sleep disturbance, constipation, diarrhea, appetite loss and difficulties score), and functional scale with social functioning, physical functioning, cognitive functioning and emotional functioning scores (15 items) [23]. Physical activity level was assessed through the modified Godin Leisure-Time Exercise Questionnaire, in which the weekly duration, as well as the frequency of light, moderate and vigorous activity, were reported [24].

## Results

No adverse events related to the integrated, multimodal approach were recorded during the 12 weeks of intervention. Compliance to the multimodal program was high: 83% (20/24) for exercise, 100% (6/6) for nutritional counseling and 75% (9/12) for psychological support sessions, respectively. Reasons for missing sessions were treatment-related side effects (fever) and invasive procedures (nephrostomy positioning). Exercise sessions were well tolerated, the nephrostomy bag did not interfere with the activity, and the planned progression was completed without modifications.

Results of the multimodal intervention are reported in Tables 1 and 2. Considerable improvements were observed for cardiorespiratory fitness (+ 26.3%), right handgrip strength (+ 9.1%), left handgrip strength (+ 4.5%), some parameters of upper and lower body flexibility and physical activity level (Table 1). Resting heart rate and blood pressure remained stable. Despite considerable weight loss in the 6 months (∼21.4%) preceding intervention, anthropometric measures showed an increase in body weight, waist/hip values and BMI from 18.0 to 19.5 kg/m^2^. Body composition analysis revealed a considerable increment in fat-free mass (+9.2%) and an improvement in phase angle, from 2.9 to 3.8°. Nutritional status improved from a NRS-2002 score of 3 (at risk for malnutrition) at baseline to a NRS-2002 score of 2 (not at risk) at the end of the intervention. Moreover, a substantial increase in energy (+ 47%) and protein (+ 75%) intake above baseline was observed; several nutritional impact symptoms present at baseline, such as dysphagia, oral mucositis, dyspepsia, xerostomia, diarrhea, steatorrhea and abdominal bloating, disappeared after 12 weeks (Table 1). QoL improved in certain domains, such as physical functioning, emotional functioning, social functioning, appetite loss; on the contrary, some symptoms especially fatigue, nausea/vomiting, pain, dyspnea and insomnia worsened during the intervention period; overall health status remained unchanged (Table 2). A clinically relevant status of depression and distress remained stable at both baseline and postintervention time points, while anxiety improved at 12 weeks, resulting in a borderline abnormal level. However, a considerable reduction in HADS-A (from 16 to 9 points), HADS-D (from 18 to 11 points) and DT (from 8 to 4 points) were observed (Table 2).

**Table 2.** Absolute scores of patient-reported outcomes.

Measure	At baseline	Postintervention
**Quality of life (score 0–100)**		
– Physical functioning	73.3	80.0
– Role functioning	50.0	50.0
– Emotional functioning	75.0	83.3
– Cognitive functioning	83.3	83.3
– Social functioning	33.3	66.7
– Global health status	50.0	50.0
– Fatigue	55.6	66.7
– Nausea/vomiting	16.6	33.3
– Pain	33.3	50.0
– Dyspnea	33.3	66.7
– Insomnia	33.3	66.7
– Appetite loss	33.3	0.0
– Constipation	0.0	0.0
– Diarrhea	66.7	66.7
– Financial problems	33.3	33.3
**Physical activity level (min/week)**		
– Vigorous	0.0	0.0
– Moderate	0.0	0.0
– Light	210.0	420.0
**Psychological status (score 0–21)**		
– Hospital anxiety and depression scale – anxiety	16	9
– Hospital anxiety and depression scale – depression	18	11
– Distress thermometer	8	4

## Discussion

This case study highlights the fact that a multimodal intervention including exercise, nutritional and psychological support carried out by a dedicated multidisciplinary team (FORCE team) is feasible and safe during palliative chemotherapy for a cachectic patient affected by advanced PC. Indeed, no adverse events related to the intervention occurred and the compliance to the three interventions was excellent. Demonstrating the safety of an exercise program, even in the context of an aggressive oncological disease, such as PC and in a patient, who recently underwent an invasive palliative procedure, may help to overcome barriers toward physical exercise in this setting [25].

Weight loss is one of the most important factors involved in cancer cachexia [8]. Approximately 80% of PC patients present cachexia, which is also a predictor of poor outcomes throughout all disease stages. As evidenced by this case study, structured multidisciplinary assessment, counseling and intervention, resulted in a substantial (∼4.2 kg) increase in body weight [8]. It is worth noting that weight gain is an essential component of the so-called clinical benefit response, a composite end point specifically developed to evaluate treatment impact in PC [26,27] and validated as a surrogate end point for survival in this setting [28]. Moreover, exercise capacity, muscular strength and mass are prognostic factors in PC [29,30] and are often impaired due to both cachexia and cancer diagnosis [31,32]. On one side, resistance training is a potent modulator of skeletal muscles, able to increase strength and muscle mass, whereas aerobic training can control inflammatory and immune response [33]. On the other hand, adequate caloric, and especially protein, intake is a key component helping to increase or preserve muscle mass [8]. An integrated, synergistic approach can favor protein turnover and increase their skeletal muscle synthesis. Our multimodal intervention resulted in an improvement in cardiorespiratory fitness, muscular strength and mass; it helped correcting nutritional deficiencies, increasing nutritional intake to meet or exceed optimal requirements, and managing nutritional impact symptoms.

**Figure 2.**
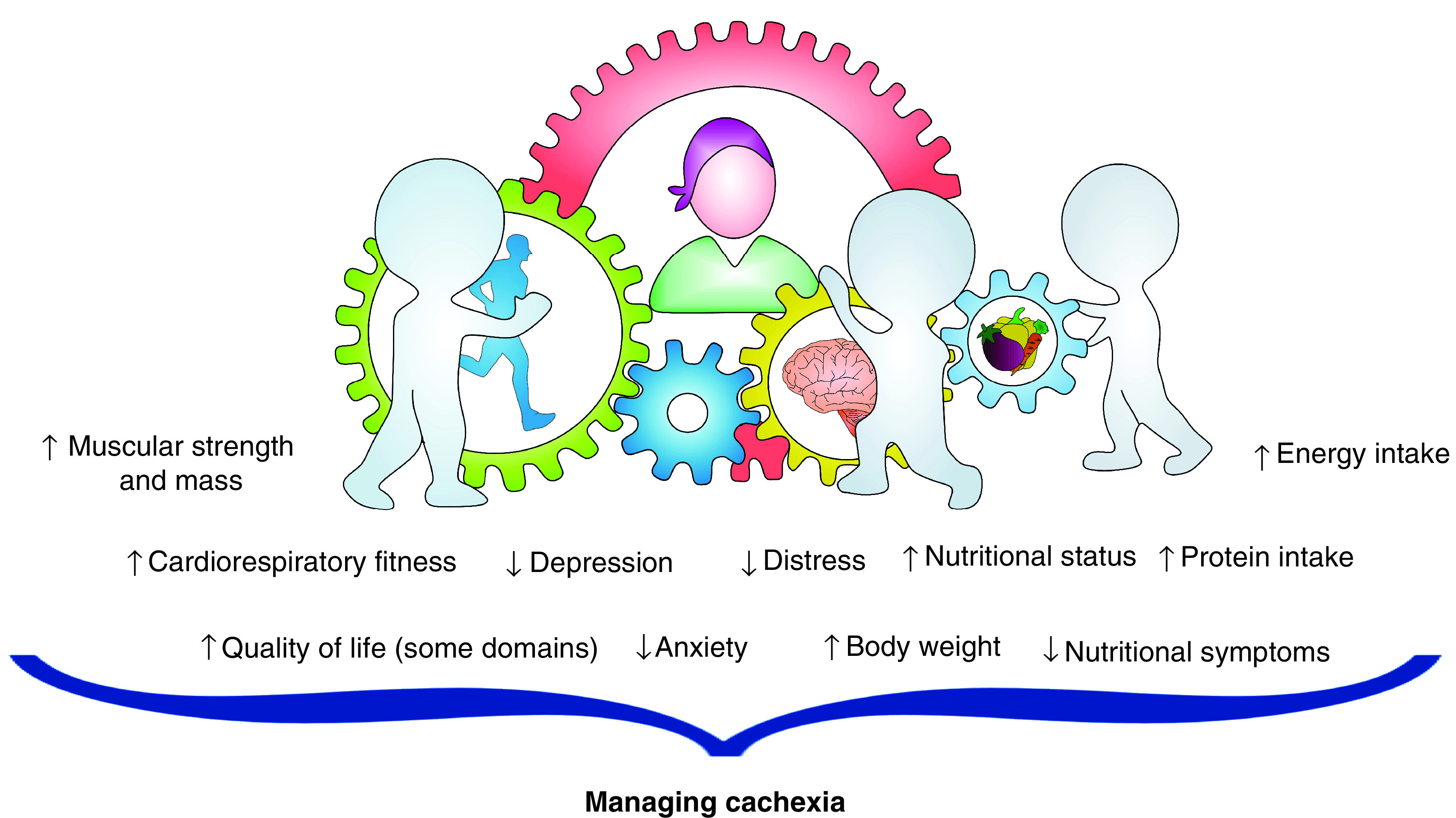
Multidisciplinary intervention as strategy to manage cancer cachexia.

Overall, it could be speculated that an increase in functional capacity, adding an adequate caloric, especially protein, intake and a gain in body weight, can improve muscle mass and consequently prevent or control cachexia (Figure 2). In addition, psychological intervention may also indirectly contribute to managing cancer cachexia. We found that distress and depression reduced substantially, and anxiety moved from abnormal levels to borderline. Consistently with a previous study [12], QoL reported improvement in some domains, while others showed no change or worsening, probably due to disease progression and change in the treatment regimen in the last two weeks of intervention. Patients with advanced cancer or with cachexia may suffer from psychological distress, and several prior investigations have identified eating-related distress as a significant contributor to these symptoms [34]. Moreover, psychological intervention can help patients to feel better, but it can also support the multimodal intervention through better uptake and adherence (Figure 2).

To the best of our knowledge, only few trials tested the feasibility of a multimodal approach for the management of cachexia. The MENAC trial evaluated in a randomized controlled design the effect of exercise, nutritional supplements and anti-inflammatory drugs, for the management of cachexia in lung and PC, showing an improvement in muscle mass and body weight, while no changes were observed for strength and exercise capacity [35]. However, compared with our case-study, the MENAC trial, reported lower levels of intervention adherence, and the program lasted only six weeks [35]. Moreover, the NEXACT intervention, including exercise, nutritional support and educative counseling, has evaluated the feasibility of a multimodal approach in elderly patients affected by pancreatic or lung cancer, reporting a good adherence to the program but, similarly, no relevant improvements in other outcomes. Although both studies had a sample size too limited to demonstrate the efficacy of an intervention, the exposure to the program and the adherence are likely to represent crucial factors to affect treatment outcome [36,37]. This may suggest that a longer intervention together with a higher adherence are needed to implement the expected results.

This analysis includes an anectoctical case of a cachectic PC patient reporting the safety and feasibility of a multidisciplinary approach. Nevertheless, some limitations should be recognized. First, our results may not be considered generalizable and definitive because it is possible that this integrated comprehensive intervention could not be feasible, safe and effective in other oncological patients with similar conditions. Second, the absence of specific blood tests could have provided additional information about the patient’s inflammatory and immunological status. In addition, appropriate measures to test the efficacy of the intervention on lower limb strength were missing and should be implemented in future studies. Finally, the integration of anticachectic agents was not considered. In this regard, the described approach may be an optimal candidate to be combined with drugs because no serious adverse events and challenges in the adherence have been reported or can be preliminary expected. Nevertheless, this case is unique for both the patient’s condition and the multimodal intervention proposed.

## Conclusion & future perspective

We found that an integrated intervention, including exercise, nutritional and psychological support, is safe, feasible and efficacious. Given the clinical relevance of the observed results, prospective trials incorporating a multimodal lifestyle approach with a solid design are strongly needed in order to validate its real benefit and definitely implement this strategy in the therapeutic management of advanced cancer.

This case report represents the backbone supporting our future research plans. A feasibility study aimed to validate the safety and feasibility of a multimodal approach to counteract cancer cachexia in PC, as well as other tumor types, is ongoing. This project will also suggest its potential efficacy on physical, nutritional and psychological end points, crucial determinants of both patients’ QoL and prognosis.

**Executive summary**

- Although relatively uncommon (2.5% of all cancers), pancreatic cancer (PC) remains a lethal malignancy.
- PC patients are at a high-risk of cachexia, a multifactorial syndrome characterized by an ongoing loss of skeletal muscle mass that cannot be fully reversed by conventional nutritional support and leads to progressive functional impairment.
- Currently, no standard treatments are available to counteract the progression of cancer cachexia.
- A 55-year-old female was diagnosed with an unresectable body/tail PC and subsequently developed liver, bone, lymph node and lung metastases.
- During chemotherapy course, a 12-week of multimodal lifestyle intervention was proposed to counteract cachexia.
- Considerable increase in body weight (4.2 kg) was observed.
- Improvements were found in cardiorespiratory fitness, muscular strength and mass, and BMI.
- The intervention improved nutritional status, increasing energy and protein intake, and reduced nutritional symptoms.
- Patient-related outcomes showed an important reduction in anxiety, depression and distress scores, while the QoL displayed improvement in some domains.
